# Case Report: KBG syndrome in an adult presenting with diabetes, gout, and hypogonadism

**DOI:** 10.3389/fendo.2026.1986666

**Published:** 2026-10-29

**Authors:** Wei Shi, Zhuhuan Xie, Chenrui Liu, Defu Kong, Nan Chen

**Affiliations:** 1 Department of Endocrinology, Anning First People’s Hospital Affiliated to Kunming University of Science and Technology, Kunming, China; 2 Faculty of Medicine, Kunming University of Science and Technology, Kunming, China

**Keywords:** ANKRD11, case report, diabetes, gout, hypogonadism, KBG syndrome, whole-exome sequencing

## Abstract

KBG syndrome (MIM #148050) is an autosomal dominant disorder caused by pathogenic variants in *ANKRD11* or 16q24.3 microdeletions, classically recognized in children through macrodontia, short stature, skeletal anomalies, and neurodevelopmental delay. How the syndrome presents to adult internal medicine remains poorly documented. A 31-year-old man was admitted with new-onset diabetes (blood glucose 20.0 mmol/L) and an acute gout flare, superimposed on congenital sensorineural deafness, repaired cryptorchidism, and 10 years of recurrent gout. Examination revealed macrodontia of the upper central incisors, kyphoscoliosis, brachydactyly with fifth-finger clinodactyly, bilateral gynecomastia, and small soft testes. Investigations showed preserved β-cell reserve with delayed insulin and C-peptide peaks, negative islet autoantibodies, repeatedly borderline-low testosterone with inadequately normal gonadotropins, and 24-h urinary uric acid excretion at the lower end of the reference range. Maternally inherited diabetes and deafness was initially suspected because diabetes coexisted with deafness, but the burden of congenital anomalies prompted trio whole-exome sequencing, which identified a *de novo* heterozygous *ANKRD11* frameshift (NM_013275.6: c.1903_1907del, p.Lys635Glnfs*26). He was treated with liraglutide, febuxostat, colchicine, and atorvastatin. Over 33 months of follow-up, glycated hemoglobin remained between 4.5% and 5.3% and thyroid function normalized, yet serum urate stayed markedly elevated at nearly every routine measurement, and one gout flare required readmission. This case illustrates delayed recognition of KBG syndrome during adulthood: the characteristic dental, skeletal, and developmental signs had been present since early childhood, yet the syndrome was named only when an acquired metabolic cluster—diabetes, gout, and hypogonadism—brought him to adult endocrine care. The decade of gout preceding glucose intolerance is, to our knowledge, the first such report in KBG syndrome and remains a single observation; whether it reflects an intrinsic urate-handling defect is a hypothesis that merits further study rather than an established association. Careful attention to dental, skeletal, and developmental clues in atypical diabetes can end a long diagnostic odyssey.

## 1 Introduction

KBG syndrome (Mendelian Inheritance in Man (MIM) #148050) is a rare autosomal dominant disorder first described in 1975 (2) and defined molecularly in 2011 by loss-of-function variants in *ANKRD11* at 16q24.3 (1, 3); the encoded chromatin-associated coregulator is essential for craniofacial, dental, skeletal, and neural development (4, 5). Macrodontia of the permanent upper central incisors (85%–95% of patients) is the most recognizable sign; hearing impairment (25%–31%), postnatal short stature, and variable intellectual disability complete the classic picture (3, 4, 6).

Two features of the literature stand out. First, it is overwhelmingly pediatric: the largest series—340 patients—centers on early life, and adult internal-medicine problems are scarcely described (4, 7). Second, endocrine and metabolic aspects beyond growth and feeding remain largely uncharted in adults (3, 7).

We encountered an adult whose KBG syndrome declared itself not in a genetics clinic but on an endocrinology ward, through new-onset diabetes, a decade of gout, and hypogonadism with gynecomastia. His is a story of delayed recognition rather than adult-onset disease: every cardinal feature was present from childhood, yet the signs were assembled into a single diagnosis only at the age of 31. We searched PubMed/MEDLINE, Embase, Web of Science, and China National Knowledge Infrastructure (CNKI) from inception to September 2026, combining “KBG syndrome” or “ANKRD11” with “adult,” “diabetes,” “hyperuricemia,” “gout,” or “hypogonadism,” without language restriction. We highlight the adult face of the syndrome and the diagnostic yield of careful physical examination in atypical diabetes. This report follows the CAse REport (CARE) guidelines (8).

## 2 Case description

### 2.1 Patient information

A 31-year-old man, a farmer from Yunnan Province, China, was admitted to our endocrinology department on 14 November 2023. Two weeks earlier he had developed marked thirst (approximately 3,000 mL daily), polyuria with nocturia, dizziness, and nausea; a local hospital recorded a blood glucose of 20.0 mmol/L (local reference range not documented) and prescribed metformin and acarbose. Ten days before admission his right ankle became red, swollen, and painful; local treatment for presumed gout brought little relief. Because his diabetes coexisted with congenital deafness, he was referred with a working diagnosis of “mitochondrial diabetes.” He mentioned recently losing his morning erections.

Bilateral cryptorchidism had been repaired at the age of four, and hearing loss was identified at six; he has worn hearing aids since. Recurrent gouty arthritis had troubled him for >10 years, self-managed irregularly with allopurinol, sodium bicarbonate, and diclofenac; the cumulative duration of allopurinol use and the date of the last dose before admission were not documented. School performance was average, and the family described long-standing social-communication difficulties, although no formal neurodevelopmental diagnosis had been established. His parents are unaffected; a grandfather and an uncle have type 2 diabetes. He neither smokes nor drinks. The clinical timeline is summarized in **Table 1**.

**Table 1:** Timeline of clinical events, diagnosis, and management.

Time point	Event
Age 4 years	Bilateral cryptorchidism surgically repaired
Age 6 years	Sensorineural hearing loss identified; hearing aids fitted
~2013 (age ~21 years)	First gout attack; recurrent flares over the following decade, self-managed irregularly with allopurinol, sodium bicarbonate, and diclofenac
2–10 November 2023	Admission to a local rehabilitation hospital; hyperuricemia, hyperglycemia, and abnormal liver enzymes recorded without a unifying diagnosis
14 November 2023	Admission to endocrinology: macrodontia, kyphoscoliosis, brachydactyly with fifth-finger clinodactyly, gynecomastia, small soft testes; acute arthritis of the right ankle
14–22 November 2023	OGTT and hormonal evaluation; abdominal, scrotal, renal, and thyroid ultrasonography; 24-h urine collection completed before urate-lowering therapy; karyotype 46,XY
14–22 November 2023	Methylprednisolone IV ×3 days and etoricoxib; febuxostat 20 mg/day plus colchicine 0.5 mg/day from 16 November; atorvastatin 20 mg/night; short-term insulin
23 November 2023	Discharge on febuxostat, colchicine, atorvastatin, and liraglutide; quarterly outpatient review scheduled
1 and 22 December 2023	Trio whole-exome sequencing and full-length mitochondrial genome sequencing received by the laboratory
12 January 2024	Molecular diagnosis: *de novo* heterozygous pathogenic ANKRD11 c.1903_1907del (p.Lys635Glnfs*26), Sanger-confirmed; mitochondrial genome negative
20 March 2024	Outpatient record first notes a congenital malformation syndrome (Q89.7); metformin reintroduced
25 April 2024	First follow-up visit under the diagnosis of KBG syndrome; febuxostat uptitration advised
December 2023–August 2026	Fifteen endocrine outpatient reviews; benzbromarone added (April 2025) and fenofibrate added (October 2025); excellent glycemic control but persistent hyperuricemia
5–9 July 2025	Gout flare; readmission to the pain department; dual-energy CT showed large knee effusions without definite urate crystals; therapeutic arthrocentesis; synovial fluid culture sterile; crystal microscopy not performed
March 2026	Thyroid function normalized without thyroid-directed treatment; vitamin D deficiency treated; the patient reported having stopped all medications about six months earlier
18 August 2026 (33 months)	Most recent review: persistent hyperuricemia and transaminase elevation; mild thrombocytopenia noted

### 2.2 Clinical findings

On examination he was of average build (168 cm, 70 kg; body mass index 24.8 kg/m²) with central adiposity (waist circumference 89 cm; waist-to-hip ratio 0.96). The most striking finding was macrodontia of the upper central incisors (**Figure 1A**); he also had mild kyphoscoliosis, brachydactyly with fifth-finger clinodactyly (**Figure 1C**), bilateral breast enlargement without nodules or discharge (**Figure 1B**), sparse axillary hair, small, soft testes (6 mL left, 8 mL right), and a flaccid penile length of 9 cm. His right ankle was warm, swollen, and tender, with limited movement. Deafness was evident during the interview. Screening for diabetic peripheral neuropathy was negative.

**Figure 1:**
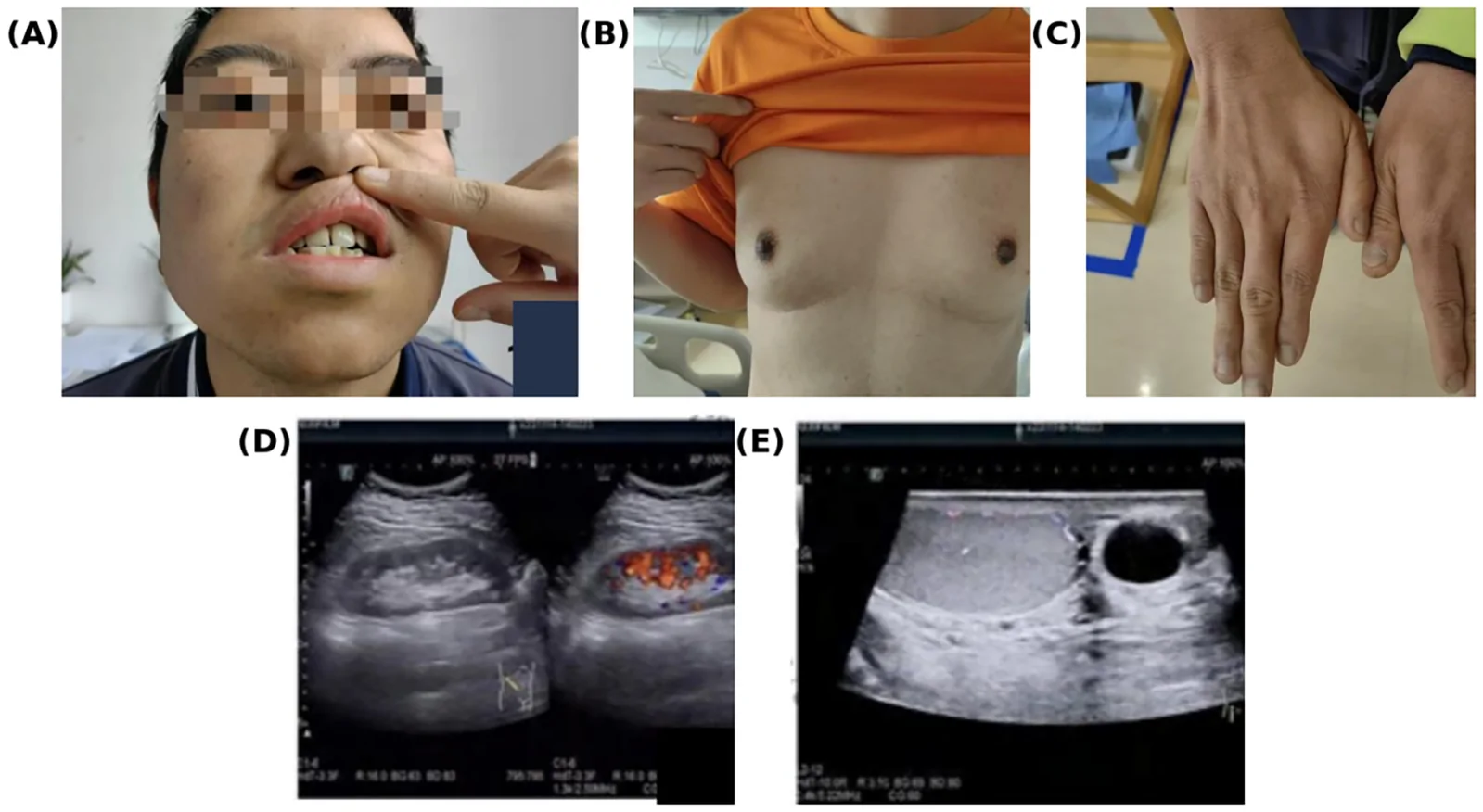
Clinical and imaging findings. **(A)** Macrodontia of the upper central incisors. **(B)** Bilateral gynecomastia without nodules or discharge. **(C)** Brachydactyly and fifth-finger clinodactyly. **(D)** Renal ultrasonography showing a 7.2 mm × 6.0 mm simple cyst in the right kidney. **(E)** Scrotal ultrasonography showing small testes, with a cyst at the lower pole of the right testis and several adjacent cystic lesions.

### 2.3 Investigations

On admission, random glucose was 6.5 mmol/L (reference 3.9–6.1) on metformin and acarbose. Urinalysis showed glucosuria (3+) and ketonuria (1+ to 2+) that cleared within 2 days. A 75-g oral glucose tolerance test showed fasting and 2-h glucose of 8.34 and 22.1 mmol/L (reference 3.9–6.1 and <7.8, respectively), with insulin and C-peptide peaking late at 3 h—insulin resistance with delayed secretion rather than absolute deficiency (**Supplementary Table 1**). HbA1c was 6.8% (reference 3.6%–6.0%), and all five islet autoantibodies were negative (**Table 2**).

**Table 2:** Key laboratory findings during hospitalization.

Parameter	Result	Reference range
Fasting plasma glucose (mmol/L)	8.3 ↑	3.9–6.1
2-h post-load glucose (mmol/L)	22.1 ↑	<7.8
HbA1c (%)	6.8 ↑	3.6–6.0
Fasting insulin (μIU/mL)	19.3 ↑	2.3–11.8
Fasting C-peptide (ng/mL)	3.63	0.78–5.19
Serum urate, outside hospital (μmol/L)	576.5 ↑	208–428
Serum urate, on admission (μmol/L)	325	208–428
24-h urinary uric acid (μmol/24 h)	2,480 (low-normal)	1,200–5,900
Testosterone (ng/mL)	2.43 ↓ (3 tests: 2.43–2.77)	2.49–8.36
LH (IU/L)	3.2–10.6 (2 tests)	1.7–8.6
FSH (IU/L)	5.3–7.0 (2 tests)	1.5–12.4
Estradiol (pmol/L)	127–144 (2 tests)	41.4–159
Prolactin (ng/mL)	14.3	4.0–15.2
Sex hormone–binding globulin (nmol/L)	5.47 ↓	13.6–76.3
Free T3 (pmol/L)	3.55 ↓	3.8–5.9
Free T4 (pmol/L)	15.85	12.0–22.0
TSH (mIU/L)	0.55 ↓	0.75–5.6
LDL cholesterol (mmol/L)	3.43 ↑	<3.37
hs-CRP (mg/L)	99.1 ↑	0–8.2
Fibrinogen (g/L)	7.83 ↑	2.0–4.0

Complete blood count, coagulation profile (apart from fibrinogen), hepatic and renal function, 24-h urinary free cortisol, islet autoantibodies (GAD, IA-2, insulin, ICA, ZnT8), and thyroid autoantibodies (TPOAb, TgAb) were within reference ranges or negative. Admission urinalysis showed glucosuria (3+) and ketonuria (1+ to 2+) that cleared within two days. The full panel, including the complete oral glucose tolerance test and adrenal steroid profile, is provided in **Supplementary Table 1**.

Serum urate had been 576.5 μmol/L 11 days earlier but measured 325 μmol/L on admission (reference 208–428 μmol/L), probably reflecting intermittent allopurinol use. A 24-h urine collection (2,500 mL), completed on 16 November 2023 at 16:34—before the first febuxostat dose (17:10) and any in-hospital urate-lowering therapy—contained 2,480 μmol of uric acid (417 mg/24 h), at the lower end of the reference range (1,200–5,900 μmol/24 h). Testosterone was repeatedly borderline-low (2.43–2.77 ng/mL; reference 2.49–8.36), while gonadotropins were not persistently elevated; estradiol and prolactin were unremarkable (**Table 2**). Sex hormone–binding globulin was low (5.47 nmol/L; reference 13.6–76.3). Androstenedione, dihydrotestosterone, cortisol, and corticosterone were likewise below range, although 24-h urinary free cortisol was normal. Free T3 (3.55 pmol/L; reference 3.8–5.9) and TSH (0.55 mIU/L; reference 0.75–5.6) were low with normal free T4 and negative thyroid autoantibodies. Inflammatory markers were elevated, consistent with the acute gout flare; LDL cholesterol was 3.43 mmol/L (reference <3.37).

Renal ultrasonography showed a 7.2 mm × 6.0 mm simple cyst in the right kidney (**Figure 1D**). Scrotal ultrasonography confirmed small testes (right 37.8 mm × 16.2 mm; left 33.1 mm × 19.1 mm) with small testicular and paratesticular cysts (**Figure 1E**). Abdominal scanning revealed fatty liver and gallbladder polyps. Echocardiography and thyroid and carotid ultrasonography were unremarkable.

### 2.4 Genetic testing

After discharge, trio whole-exome sequencing of the patient and his parents (KingMed Center for Clinical Laboratory, Guangzhou, China; mean depth ≥90×, approximately 98% of target bases at ≥20×) identified a heterozygous ANKRD11 frameshift variant (NM_013275.6: c.1903_1907del, p.Lys635Glnfs*26; exon 9 of 13), confirmed by Sanger sequencing; both parents were wild type, indicating a *de novo* event (**Figure 2**). Per the American College of Medical Genetics and Genomics (ACMG)/Association for Molecular Pathology (AMP) guidelines (9, 10), the variant—absent from Genome Aggregation Database (gnomAD), previously reported as pathogenic in unrelated patients with KBG syndrome (ClinVar VCV000496332), and predicted to trigger nonsense-mediated mRNA decay—was classified as pathogenic. No explanatory copy-number variants were detected, and the karyotype was 46,XY. Given the admitting suspicion of mitochondrial disease, the full-length mitochondrial genome was amplified by long-range PCR and sequenced against the revised Cambridge Reference Sequence (NC_012920.1); no pathogenic or likely pathogenic variant was detected, although sensitivity for large-scale deletions is limited.

**Figure 2:**
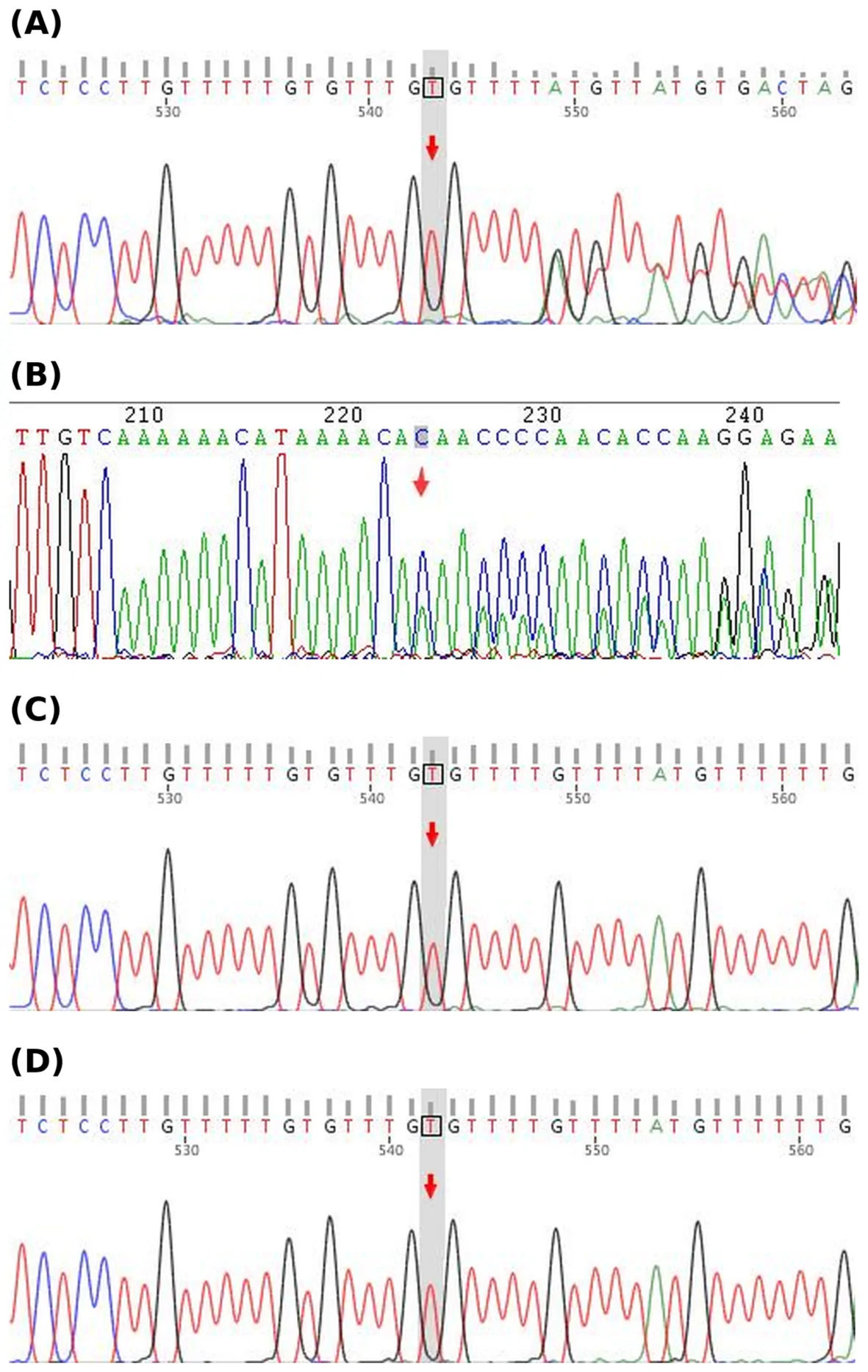
Sanger sequencing validation of the *ANKRD11* c.1903_1907del (p.Lys635Glnfs*26) variant. **(A)** Proband, forward strand: overlapping peaks downstream of the deletion breakpoint, indicating a heterozygous frameshift deletion. **(B)** Proband, reverse strand. **(C)** Father, forward strand: wild type. **(D)** Mother, forward strand: wild type. Red arrows indicate the deletion site.

## 3 Diagnostic assessment

The syndromic differential was narrowed by the phenotype itself: Cornelia de Lange syndrome lacked synophrys, limb-reduction defects, or significant growth retardation (11); Silver–Russell syndrome lacked intrauterine growth retardation, relative macrocephaly, or body asymmetry (12); and Aarskog–Scott syndrome was incompatible with the *de novo* inheritance and dental phenotype (13). A 16q24.3 microdeletion was excluded by copy-number analysis (14). The 2016 clinical diagnostic criteria were fulfilled (7).

Diabetes classification required equal care. Type 1 diabetes was excluded by negative autoantibodies and preserved, delayed-peak C-peptide despite admission ketonuria. Maternally inherited diabetes and deafness—the admitting suspicion—was rendered unlikely by absent maternal transmission and full-length mitochondrial genome sequencing (15), and maturity-onset diabetes of the young by the negative exome story (16). Common type 2 diabetes with central adiposity and insulin resistance best fits the data. The monoarticular ankle arthritis with prior hyperuricemia and a prompt response to anti-inflammatory therapy was diagnosed as a gout flare (17).

## 4 Therapeutic intervention

For the acute gout flare, intravenous methylprednisolone was given for 3 days with etoricoxib 60 mg daily. Febuxostat 20 mg daily and colchicine 0.5 mg daily were started after completion of the 24-h urine collection (18), and atorvastatin 20 mg nightly was added for dyslipidemia. In-hospital hyperglycemia was managed with short-term insulin. Liraglutide was initiated at 0.6 mg daily and up-titrated to 1.8 mg over 2 weeks, with diabetes education and dietary counseling (19). Metformin was withheld at discharge because a mitochondrial etiology had not yet been excluded, and allopurinol was not continued. Quarterly outpatient review was scheduled. Hearing aids were continued.

## 5 Follow-up and outcomes

He was discharged on 23 November 2023 free of joint pain, with much improved glycemic control; the molecular diagnosis arrived 7 weeks later. Fifteen endocrine outpatient encounters and one readmission were documented over the following 33 months, up to 18 August 2026 (**Table 1**). Glycemic control was excellent throughout (glycated hemoglobin 4.5%–5.3% across eight reviews), and thyroid function had normalized by March 2026 (free T3 4.39 pmol/L, TSH 3.48 mIU/L). Serum urate, in contrast, remained markedly elevated at nearly every routine measurement—637, 643, 574, 619, 538, 587, 567, 693, 569, and 513 μmol/L—despite prescribed febuxostat, dose up-titration advised in April 2024, and benzbromarone added in April 2025, a persistence that underscores the need for strict titration and adherence monitoring to reach target urate (<360 μmol/L) (18); the single in-range reading (427 μmol/L) was drawn during a flare readmission, when serum urate characteristically falls (17). In July 2025, his only gout flare of the interval required readmission to the pain department: dual-energy CT showed large knee effusions but no definite urate crystal deposition, the left knee was aspirated for symptomatic relief (35 mL of pale-yellow fluid; 7 mL at repeat aspiration; none from the right knee) followed by intra-articular betamethasone and sodium hyaluronate, and synovial fluid culture was sterile; crystal microscopy was not performed. Medications evolved: semaglutide replaced liraglutide (December 2023), metformin was reintroduced (March 2024) and paused (October 2024), fenofibrate was added (October 2025), and by March 2026 he reported having stopped all medications for approximately 6 months. Transaminase elevation was recurrent—ALT 56, 110, 71, 103, and finally 157 U/L (reference 9–50)—with mild thrombocytopenia at nearly every measurement (platelets 76–123 × 10^9^/L, once 158; reference 125–350); calculated FIB-4 scores remained indeterminate (1.36–1.97), viral hepatitis and HIV were excluded serologically, bicyclol had been started in April 2025, and hepatic injury was recorded as a formal diagnosis in July 2025. Adherence was documented to be poor, with prolonged self-initiated treatment gaps; body weight was 68 kg at the first outpatient review (December 2023), 2 kg below baseline, and was never recorded thereafter.

## 6 Discussion

This patient was 31 before his lifelong condition finally had a name, although the evidence—an oversized incisor, a curved spine, undescended testes, deafness—had been on display for decades: a case of delayed recognition rather than adult-onset disease. Such delay appears common rather than exceptional: in a patient- and caregiver-reported survey of 91 adults with KBG syndrome, nearly half of respondents with a known diagnosis age had been diagnosed at 18 years or older (32). The delay was not cost-free—a decade of gout and a non-diagnostic rehabilitation-hospital admission preceded recognition. Two recent studies chart the adult phenotype: Bayat et al. assembled a physician-reported cohort of 36 molecularly confirmed adults and reviewed a further 154 in the literature, finding endocrine comorbidities in 17% (6 of 36), including type 2 diabetes diagnosed at age 30 and autoimmune hypothyroidism in three adults (33); Low et al. described high rates of psychiatric, behavioral, sleep, seizure, hearing, visual, dental, and skeletal problems, with a higher-than-expected cardiovascular and gastrointestinal burden (32). Gout was not reported in either cohort, and diabetes and gout were likewise absent from the large pediatric-focused series (4, 7); series from Europe and China focus on children and adolescents (20, 21). To our knowledge, the conjunction of KBG syndrome with this metabolic cluster has not previously been reported.

The admitting diagnosis—maternally inherited diabetes and deafness—was reasonable on its face: diabetes with sensorineural deafness is its textbook pairing, and the m.3243A>G mutation is a recognized cause in adults (15). Three observations argued against it: congenital rather than progressive deafness, paternal family history, and congenital anomalies exceeding what mitochondrial disease explains. Careful physical examination proved decisive: macrodontia, clinodactyly, and repaired cryptorchidism redirected the work-up toward a syndromic disorder, and trio whole-exome sequencing settled the question (9). Recognition preceded sequencing—KBG syndrome in older patients is readily missed unless deliberately sought (7, 20).

Testosterone was borderline-low across three measurements, yet gonadotropins did not rise as compensated primary testicular failure would predict—an inadequate response suggesting a central contribution. The phenotype is most consistent with partial hypogonadotropic hypogonadism, provisional pending dynamic confirmation, although a mild testicular component remains plausible given the repaired cryptorchidism and small testicular volumes. Hypogonadism and the metabolic phenotype are plausibly interconnected through adiposity: central obesity and insulin resistance suppress sex hormone–binding globulin (low at 5.47 nmol/L; reference 13.6–76.3) and thereby total testosterone, while hypogonadism favors fat accumulation in turn; any direct contribution of *ANKRD11* is unknown. Gonadal involvement is not foreign to KBG syndrome—cryptorchidism, micropenis, and pubertal abnormalities recur in published series (22, 23)—and his gynecomastia fits the same hormonal logic: the molar testosterone-to-estradiol ratio was approximately 66, well below that of healthy adult men, and a reduced androgen-to-estrogen balance is an established mechanism of male breast development (24, 25). Other pituitary axes showed no deficiency: growth hormone was within the reference range, insulin-like growth factor 1 was marginally above the upper reference limit (327.9 ng/mL; age-specific reference 115–320 ng/mL), 24-h urinary free cortisol was normal, an extended adrenal steroid panel 4 months later was unremarkable, thyroid function normalized spontaneously, and the karyotype was 46,XY. Pituitary imaging, dynamic testing (GnRH and hCG stimulation), inhibin B, and semen analysis were not undertaken; the axis cannot be localized with certainty (see *Limitations*).

The ten-year gout history, preceding diabetes by a decade, is the most provocative feature: attributing the hyperuricemia to diabetic insulin resistance would be backwards—gout came first. His 24-h urinary uric acid excretion (2,480 μmol) sat at the lower end of the reference range despite a controlled hospital diet—consistent with relatively low excretion rather than overproduction—although a formal fractional excretion could not be calculated because urinary creatinine was not measured. Serum urate remained above range at nearly every routine measurement over 33 months despite prescribed therapy. This persistence is plausibly explained by conventional, potentially confounding factors—central adiposity, insulin resistance, steatotic liver disease, dyslipidemia, suboptimal dose titration, and documented poor adherence—rather than by the *ANKRD11* variant itself, although intrinsically severe hyperuricemia cannot be excluded. Gout has not been reported in the adult KBG cohorts to date (32, 33); we therefore present the association as a single observation that generates a hypothesis, not as evidence of an expanded phenotype. A population-level hint exists: in an epigenome-wide association study of serum urate, *ANKRD11* was implicated by urate-associated cytosine–phosphate–guanine (dinucleotide) (CpG)s and showed reduced renal expression in a humanized mouse model of hyperuricemia (26)—although such differences may lie downstream rather than upstream. Whether a chromatin coregulator touches the tubular urate-transport machinery (27) is a question for the laboratory, not for this report.

His diabetes is ordinary in mechanism if unusual in company: central adiposity, a delayed insulin peak with sustained hyperinsulinemia, negative autoantibodies, and type 2 diabetes in paternal relatives. Whether KBG syndrome adds independent risk cannot be answered from one case, although type 2 diabetes and autoimmune thyroid disease in the physician-reported adult cohort (33) suggest that metabolic surveillance of adults with KBG syndrome may be worthwhile. The mechanistic literature offers indirect support—*ANKRD11* haploinsufficiency reshapes transcriptional programs in developing mouse tissues (6), and the encoded protein coregulates nuclear receptors central to metabolic control (5)—and no direct evidence links *ANKRD11* to insulin signaling, adipogenesis, or β-cell development; we present the association as an observation for future adult series.

The thyroid profile likewise invites restraint: low free T3 with low TSH and normal free T4 during an acute gout flare is the classic signature of non-thyroidal illness rather than central hypothyroidism, in which free T4 would fall (28); by follow-up, free T3 and TSH normalized without thyroid-directed treatment. The low serum cortisol and corticosterone with normal 24-h urinary free cortisol are best read the same way—an acute-illness artifact rather than adrenal insufficiency.

Three lessons follow. First, diabetes with deafness is not automatically mitochondrial disease—Wolfram and Alström syndromes and mitochondrial deletion disorders occupy the same territory (29–31), and this case adds KBG syndrome to that list. Second, in atypical diabetes, careful attention to teeth and hands may be more informative than an expanded autoantibody panel. Third, whole-exome sequencing delivers most when pointed by a phenotype (9).

This report has limitations. It describes a single patient, and causality cannot be established. Urinary creatinine was not measured in the 24-h collection (completed before the first febuxostat dose), so a formal fractional excretion of uric acid could not be calculated. Pituitary MRI, dynamic pituitary–gonadal testing, inhibin B, and semen analysis were unavailable, so the hypogonadism cannot be localized with certainty. Synovial fluid crystal microscopy was not performed during the 2025 flare, and dual-energy CT showed no definite crystal deposition; the diagnosis of gout rests on clinical criteria. Formal neuropsychological assessment was never performed. Hepatic fibrosis was assessed only indirectly through FIB-4, whose validation is limited below 35 years of age; elastography was unavailable. Body weight was not systematically recorded during follow-up, and the pre-treatment gout-attack frequency could not be reconstructed. Finally, blood-based mitochondrial sequencing neither reliably detects large-scale deletions nor excludes tissue-specific heteroplasmy, so a mitochondrial contribution, although unlikely, cannot be excluded. We present the endocrine and metabolic associations as hypotheses anchored to the literature, not as demonstrated mechanisms.

## 7 Patient perspective

“For more than ten years I thought gout was my only problem. I never imagined that my teeth, my hearing, and the operation I had as a small child could be parts of one illness. When the doctors told me there was a name for it, and that it was no one’s fault, I felt relieved for the first time. I now take my medicine and return for checks every three months. I agreed to share my story and photographs so others like me might be found earlier.” (translated from Mandarin).

His account mirrors the theme of this report: each feature had been named separately—bad teeth, deafness, gout, diabetes—but no one had read them as one story. His relief at a unifying diagnosis echoes the lived experience of other adults with KBG syndrome (32) and shows why timely recognition matters to patients as much as to clinicians.

## 8 Conclusion

KBG syndrome may escape recognition until adult life, when an acquired metabolic cluster—diabetes, gout, and hypogonadism—can overshadow dental, skeletal, and developmental signs present since childhood. Diabetes has been documented in adults with KBG syndrome and may justify metabolic surveillance; the gout that preceded diabetes by a decade, with hyperuricemia persisting through 33 months of follow-up, remains a single observation generating a testable hypothesis rather than evidence of an expanded phenotype. In adults with atypical diabetes, deliberate attention to dental, skeletal, and developmental clues can shorten a long diagnostic odyssey.

## Data Availability

The original contributions presented in the study are included in the article/**Supplementary Material**. Further inquiries can be directed to the corresponding author.
