# Supplementary material for: Case Report: KBG syndrome in an adult presenting with diabetes, gout, and hypogonadism
Source: Front Endocrinol (Lausanne). 2026 Oct 29;17:1986666. doi: 10.3389/fendo.2026.1986666 (PMC13754706; doi:10.3389/fendo.2026.1986666)
Supplement: Supplementary file 1 [file Table1.docx]

**Supplementary Table 1.** Extended laboratory panel.

| **Parameter** | **Result** | **Reference range** |
| --- | --- | --- |
| **OGTT (75 g) — glucose (mmol/L)** |  |  |
| 0 / 0.5 / 1 / 2 / 3 h | 8.34 / 15.12 / 17.70 / 22.10 / 16.76 | fasting 3.9–6.1 |
| **OGTT — insulin (μIU/mL)** |  |  |
| 0 / 0.5 / 1 / 2 / 3 h | 19.3 / 34.20 / 38.80 / 68.60 / 79.50 | fasting 2.3–11.8 |
| **OGTT — C-peptide (ng/mL)** |  |  |
| 0 / 0.5 / 1 / 2 / 3 h | 3.63 / 5.82 / 6.53 / 11.27 / 12.25 | fasting 0.78–5.19 |
| **Sex hormones, 15 Nov 2023** |  |  |
| LH / FSH (IU/L) | 10.6 ↑ / 7.0 | 1.7–8.6 / 1.5–12.4 |
| Testosterone (ng/mL) | 2.57 | 2.49–8.36 |
| Estradiol (pmol/L) | 144 | 41.4–159 |
| Prolactin (ng/mL) | 13.9 | 4.0–15.2 |
| **Sex hormones, 22 Nov 2023** |  |  |
| LH / FSH (IU/L) | 3.2 / 5.3 | 1.7–8.6 / 1.5–12.4 |
| Testosterone (ng/mL) | 2.43 ↓ | 2.49–8.36 |
| Estradiol (pmol/L) | 127 | 41.4–159 |
| Prolactin (ng/mL) | 14.3 | 4.0–15.2 |
| **Sex hormone–binding globulin, 15 Nov 2023 (nmol/L)** | 5.47 ↓ | 13.6–76.3 |
| **Adrenal steroid profile, 15 Nov 2023** |  |  |
| Testosterone (ng/mL) | 2.77 | 2.5–11.0 |
| Androstenedione (ng/mL) | 0.30 ↓ | 0.40–2.20 |
| Dihydrotestosterone (ng/mL) | 0.04 ↓ | 0.16–0.79 |
| DHEA-S (ng/mL) | 1,573 | 50–6,900 |
| Cortisol (ng/mL) | 7 ↓ | 60–260 |
| Corticosterone (ng/mL) | 0.17 ↓ | 0.6–12.93 |
| Cortisone (ng/mL) | 1 ↓ | 12–35 |
| Aldosterone (ng/mL) | 0.14 | 0.03–0.16 (supine) |
| 17α-hydroxyprogesterone (ng/mL) | 0.48 | ≤3.07 |
| **Thyroid function** |  |  |
| Free T3 / Free T4 (pmol/L) | 3.55 ↓ / 15.85 | 3.8–5.9 / 12.0–22.0 |
| TSH (mIU/L) | 0.55 ↓ | 0.75–5.6 |
| TPOAb / TgAb | negative / negative |  |
| **24-h urine studies, 16 Nov 2023** |  |  |
| Urine volume (mL) | 2,500 |  |
| Urinary uric acid (μmol/L; μmol/24 h) | 992; 2,480 | 1,200–5,900 μmol/24 h |
| Note | Urinary creatinine was not measured in this collection, so the fractional excretion of uric acid (FEUA) could not be calculated. The collection was completed on 16 November 2023 at 16:34, before the first dose of febuxostat (17:10). | — |
| Urinary free cortisol (μg/24 h) | 295.5 | 50–437 |
| **Inflammatory markers** |  |  |
| hs-CRP (mg/L) | 99.1 ↑ | 0–8.2 |
| Procalcitonin (ng/mL) | 0.21 ↑ | <0.1 |
| Fibrinogen (g/L) | 7.83 ↑ | 2.0–4.0 |
| **Other** |  |  |
| LDL cholesterol (mmol/L) | 3.43 ↑ | <3.37 |
| 25-hydroxyvitamin D (ng/mL) | 30 | deficiency <20; insufficiency 20–30 |
| Parathyroid hormone (pg/mL) | 52.95 | 12–88 |
| Karyotype, 23 Nov 2023 | 46,XY | — |
| Growth hormone, 15 Nov 2023 (ng/mL) | 0.06 | 0.05–2.40 |
| IGF-1, 15 Nov 2023 (ng/mL) | 327.9 ↑ | 115–320 (age 31–35 years) |
| **Serial follow-up panel** |  |  |
| 24 Apr 2024: HbA1c (%) / serum urate (μmol/L) | 5.0 / 637 ↑ | 3.6–6.0 / 208–428 |
| 16 Oct 2024: HbA1c / serum urate | 4.7 / 643 ↑ |  |
| 14 Jan 2025: HbA1c / serum urate / ALT / platelets | 4.9 / 574 ↑ / 27 / 158 | 3.6–6.0 / 208–428 / 9–50 / 125–350 |
| 15 Apr 2025: HbA1c / serum urate | 5.3 / 619 ↑ |  |
| 5 Jul 2025: HbA1c / serum urate | 4.8 / 427 | readmission for gout flare |
| 29 Jul 2025: HbA1c / serum urate | 4.5 / 538 ↑ |  |
| 28 Oct 2025: HbA1c / serum urate | 4.5 / 587 ↑ |  |
| 17 Mar 2026: HbA1c / serum urate | 4.9 / 567 ↑ |  |
| 17 Mar 2026: fasting glucose / insulin / C-peptide | 6.25 mmol/L ↑ / 5.3 μIU/mL / 2.19 ng/mL |  |
| 17 Mar 2026: free T3 / free T4 / TSH | 4.39 / 16.52 pmol/L / 3.48 mIU/L | all within range |
| 17 Mar 2026: ALT / AST / ALP (U/L) | 103 ↑ / 45 ↑ / 128 ↑ | 9–50 / 15–40 / 45–125 |
| 17 Mar 2026: triglycerides / HDL-C / LDL-C (mmol/L) | 2.36 ↑ / 1.02 ↓ / 2.57 | <1.7 / 1.16–1.42 / <3.37 |
| 17 Mar 2026: creatinine (μmol/L); eGFR | 69.9; 117.95 mL/min/1.73m² | 57–97 |
| 21 Apr 2026: serum urate (μmol/L) | 693 ↑ | 208–428 |
| 26 May 2026: serum urate (μmol/L) | 569 ↑ | 208–428 |
| 18 Aug 2026: serum urate / fasting glucose / ALT / AST / platelets | 513 ↑ / 6.48 mmol/L ↑ / 157 U/L ↑ / 62 U/L ↑ / 97 ×10⁹/L ↓ | 208–428 / 3.9–6.1 / 9–50 / 15–40 / 125–350 |
| Note | In the serial follow-up panel, dates are laboratory sampling dates; the corresponding clinic visit occurred the same or the following day (e.g., sampling on 24 April 2024 preceded the clinic visit on 25 April 2024). | — |
